# ERAS Is Constitutively Expressed in the Tissues of Adult Horses and May Be a Key Player in Basal Autophagy

**DOI:** 10.3389/fvets.2022.818294

**Published:** 2022-05-24

**Authors:** Francesca De Falco, Antonella Perillo, Fabio Del Piero, Chiara Del Prete, Nicola Zizzo, Ioan Marcus, Sante Roperto

**Affiliations:** ^1^Dipartimento di Medicina Veterinaria e Produzioni Animali, Università degli Studi di Napoli “Federico II”, Napoli, Italy; ^2^Dipartimento di Medicina Veterinaria, Università degli Studi di Bari “Aldo Moro”, Bari, Italy; ^3^Department of Pathobiological Sciences and Louisiana Animal Disease Diagnostic Laboratory-LADDL, School of Veterinary Medicine, Louisiana State University, Baton Rouge, LA, United States; ^4^Pathology Department, Faculty of Veterinary Medicine, University of Agricultural Sciences and Veterinary Medicine, Cluj-Napoca, Romania

**Keywords:** adult horse, basal autophagy, constitutive expression, ERas, mitophagy

## Abstract

ERas is a new gene of the Ras family found in murine embryonic stem (ES) cells. Its human ortholog is not expressed in human ES cells. So far ERas gene has only been found to be expressed in the tissues of adult cynomolgus monkeys and cattle; however, information about ERAS expression or its potential functions in equine tissues is lacking. This study was performed to investigate whether Eras is an equine functional gene and whether ERAS is expressed in the tissues of adult horses and determine its potential physiological role. Expression of the ERas gene was detected in all examined adult tissues, and the RT-PCR assay revealed ERAS transcripts. Protein expression was also detected by Western blot analysis. Quantitative real time RT-qPCR analysis revealed that different expression levels of ERAS transcripts were most highly expressed in the testis. Immunohistochemically, ERAS was found to be localized prevalently in the plasmatic membrane as well as cytoplasm of the cells. ERAS was a physical partner of activated PDGFβR leading to the AKT signaling. ERAS was found to interact with a network of proteins (BAG3, CHIP, Hsc70/Hsp70, HspB8, Synpo2, and p62) known to play a role in the chaperone-assisted selective autophagy (CASA), which is also known as BAG3-mediated selective macroautophagy, an adaptive mechanism to maintain cellular homeostasis. Furthermore, ERAS was found to interact with parkin. PINK1, BNIP3, laforin. All these proteins are known to play a role in parkin-dependent and -independent mitophagy. This is the first study demonstrating that Eras is a functional gene, and that ERAS is constitutively expressed in the tissues of adult horses. ERAS appears to play a physiological role in cellular proteostasis maintenance, thus mitigating the proteotoxicity of accumulated misfolded proteins and contributing to protection against disease. Finally, it is conceivable that activation of AKT pathway by PDGFRs promotes actin reorganization, directed cell movements, stimulation of cell growth.

## Introduction

Embryonic Ras (ERas) is a novel member of the Ras family, that was first identified in murine embryonic stem (ES) cells. It is localized on the X chromosome and encodes a small GTPase protein composed of 227 amino acids that shared 43, 46, and 47% identity to the conventional Ras oncogenes H-ras, K-ras, and N-ras, respectively (1). Unlike other proteins of the Ras family, ERAS is constitutively active without any mutations (1, 2). Expressed sequence tag (EST) databases indicate that orthologs of this gene are expressed in other mammals (3). A truncated noncoding ERas transcript has been identified in human ES cells resulting from a premature polyadenylation signal upstream of its coding sequence (3). By *in vivo* studies, ERAS was found to be expressed both in ES cells and in tissues of adult cynomolgus monkeys, Asian long-tailed macaques (*Macaca fascicularis*) (4), and in tissues of adult cattle (5, 6). It has been shown that ERAS modulates the Akt signaling pathway and, thus appears to be involved in promoting cell proliferation and tumorigenicity. It has been suggested that ERAS coordinates cell proliferation and cell differentiation, plays a central role in the stimulation of somatic cell reprogramming, which is similar to the cancer initiation process (7, 8). Eras regulates epithelial-mesenchymal transition in pancreatic cancer cells *via* the ERK/AKT signaling pathway (9). ERAS expression has been found in certain human cancers, including colorectal, pancreatic, neuroblastoma, and breast carcinoma cell lines, as well as in mouse mammary tumors (10–13). ERAS is also expressed in human gastric cancer, where it may play a crucial role in gastric cancer cell survival and metastasis to the liver *via* downregulation of E-cadherin (14, 15). Recently, ERAS overexpression appeared to have strong oncogenic ability in triggering human colorectal cancer due to its ability to activate AKT signaling (16).

ERAS was found to be constitutively expressed in bovine placental tissue (6) and ERAS overexpression was detected in naturally occurring bladder cancer of cattle associated with bovine papillomavirus (BPV) infection (5). ERAS was found to interact physically with the activated platelet-derived growth factor β receptor (PDGFβR), thus forming a ternary complex with BPV E5 oncoprotein. This complex appeared to play a crucial role in the phosphorylation of AKT, a downstream effector of both ERAS and PDGFβR, in these tumors (5). Recently, ERAS was found to interact with a protein network involved in macroautophagy, including mitophagy induced by BPVs (17, 18).

Our goal is to provide evidence about ERAS expression in tissues of adult horses and its interaction with a network of proteins, some of them known to be interactors with the cellular autophagy machinery.

## Materials and Methods

### Tissue Samples

Tissue samples such as the medulla oblongata, pons, cerebellum, heart, lung, liver, kidneys, spleen, intestine, uterus, ovary, and testis, were collected from fifteen 3 to 5-year-old horses. All animals were from eastern Europe. Furthermore, at term placenta samples were obtained from ten 3 to 5-year-old Italian standardbred pregnant mares after placenta expulsion during physiological parturition. Tissue samples were immediately divided into several parts with some fixed in 10% buffered formalin for microscopic investigations and others immediately stored at −80 °C for subsequent molecular analysis. The remaining parts were submerged in RNA later storage reagent to stabilize and protect RNA.

### Antibodies

Antibodies against HspB8 (SC-22393), Hsc70/Hsp70 (SC-33575), carboxyl terminus of Hsc70-interacting protein (CHIP) (SC-133083), total PDGFβR (SC-432), phosphorylated PDGFβR (pPDGFβR) (SC-12909), β-actin (SC-47778), p62 (SC-28359), PTEN-induced putative kinase1 (PINK1) (SC-33796) and BNIP3 (SC-56167) obtained from Santa Cruz Biotechnology (TX, USA) were diluted 1:500 in BSA. AKT (#9272) antibody obtained from Cell Signaling (LID, NL) was diluted 1:1000 in BSA. Rabbit polyclonal anti-Bag3 (27 F 189C3) obtained from Biouniversa s.r.l. (AV, Italy) was diluted 1:2000 in BSA. Rabbit polyclonal anti-Synaptopodin 2 (Synpo2) (it) (orb453548) obtained from Biorbyt (CA, USA) was diluted 1:1000 in BSA. Antibody against laforin obtained from Novus Biologicals (NBP2-24474) (CO, USA) was diluted 1:500 in BSA. Rabbit antibody against human embryonic stem cell-expressed Ras (ERAS) (TA324562), diluted 1:1000 in BSA, with ERAS HEK 293T cell transient overexpression lysate and WB positive control (COD. LC405694) was obtained from OriGene Technologies, Inc. (MD, USA). This specific antibody was produced against a human ERAS epitope composed of 31 amino acid residues at position 51–81. The human epitope revealed a 100% identity with the same equine ERAS epitope.

### Immunohistochemistry

Tissues fixed in 10% neutral buffered formalin were progressively alcohol dehydrated and paraffin embedded. Five-micron sections from several tissues were immunolabelled using the avidin-biotin-peroxidase complex (ABC) method with the Vectastain ABC kit (Vector Laboratories, Inc., CA, USA). Tissue sections were deparaffinized in decreasing alcohol solutions and endogenous peroxidase activity was blocked by incubation in 0.3% H_2_O_2_ in methanol for 20 min. Antigen retrieval was performed by pre-treatment with microwave heating (twice for 5 min each at 700 W) in EDTA, pH 8.0. The slides were washed three times with phosphate buffered saline (PBS, pH 7.4, 0.01 M), and then incubated for 1 h at room temperature with normal goat serum (Santa Cruz Biotechnology, TX, USA) diluted at 20% in PBS. The excess serum was gently drained and a polyclonal rabbit anti-ERAS primary antibody (OriGene Technologies, Inc. MD, USA) diluted at 1:100 in PBS was applied overnight at +4°C in a humid chamber. The slides were washed three times with PBS, and then incubated for 30 min with a goat anti-rabbit biotinylated secondary antibody (Vector Laboratories Inc., CA, USA) diluted at 1:200 in PBS. Sections were washed three times with PBS and then incubated with Vectastain ABC reagent (Vector Laboratories Inc., CA, USA) for 30 min in a humid chamber at room temperature. Color development was obtained by treatment with 3, 3'-diaminobenzidine (Vector Laboratories Inc., CA, USA) for 2–10 min. Sections were counterstained with Mayer's hematoxylin. Species- and isotype-matched immunoglobulin (IgG) replaced the primary antibody in the negative controls at the same concentration.

### RNA Extraction and Reverse Transcription-Polymerase Chain Reaction

Total RNA was extracted from 15 equine tissues (brain, cerebellum, pons, intestine, lung, spleen, heart, testis, uterus, ovary, kidney, liver and placenta) using an RNeasy Mini Kit (Qiagen TM, DE), in accordance with the manufacturer's instructions. Genomic DNA was removed from the RNA samples using RNase-free DNase I from Fermentas Life Sciences (Thermo Fisher Scientific, MA, USA). One microgram of the total RNA was used to generate a single strand of cDNA using the QuantiTect Reverse Transcription Kit (Qiagen TM, DE), according to the manufacturer's instructions. PCR was performed both on samples in which reverse transcriptase was added to the reaction mix and in those without adding RT with specific primer sets designed using Primer 3, an online tool, for the equine ERAS: forward 5'-GTAGAAGACCACGACCCCAC-3', reverse 5'- GAAGGGTCATCGAGGGCAAA-3'; (179 bp) and ß-actin: forward 5′- TCCCTGGAGAAGAGCTACGA-3′, reverse 5′-GGATTCCATGCCCAGGAAGG-3′ (111 bp). Primers for both ERAS and ß-actin were evaluated showing a 100% efficiency. The conditions used for PCR were: 94 °C for 5 min, followed by 35 cycles of 95 °C for 30 s, 58 °C for 30 s and 72 °C for 30 s.

### Sequence Analysis

PCR products from cDNA, were purified using a Qiaquick PCR purification Kit (Qiagen TM, ME, DE) and bidirectionally sequenced using a BigDye Terminator v1.1 Cycle Sequencing Kit (Applied Biosystems, CA, USA) following the manufacturer's recommendations. Sequences were removed with a DyeEx_2.0 spin kit (Qiagen TM, DE) and run on a 3,500 Genetic Analyzer (Applied Biosystems, CA, USA). Electropherograms were analyzed using Sequencing analysis v5.2 and sequence scanner v1.0 software (Applied Biosystems, CA, USA). The sequences obtained were compared to others in GenBank using the BLAST program.

### Real Time RT-QPCR

To perform real time RT-qPCR analysis, total RNA and cDNA from 15 equine tissues were generated as reported above. Real time PCR was carried out on a BioRad CFX Connect™ Real Time PCR Detection System (Bio Rad Hercules, CA, USA) using iTAq Universal SYBR® Green Supermix (Bio Rad CA, USA). Each reaction was performed in triplicate, and the primers used for ERas and ß-actin were the same as those for RT-PCR assay. The thermal profile for the PCR was: 95°C for 10 min, 40 cycles of 94°C for 15 s, and 58°C for 30 s, followed by a melting curve. The relative quantification (RQ) was carried out using CFX Manager™ software, based on the equation RQ=2^−Δ*Cq*^, where Cq is the quantification cycle to detect fluorescence. Cq data were normalized to the equine β-actin gene (forward: 5′-TCCCTGGAGAAGAGCTACGA-3′, reverse 5′-GGATTCCATGCCCAGGAAGG-3′).

### Western Blot

Western blot analysis was performed on 15 equine tissues. The tissue samples were lysed in RIPA assay-morpholinepropanesulfonic acid (RIPA-MOPS) buffer (20 mM MOPS, 150 mM NaCl, 1 mM EDTA, 1% NP-40, 1% deoxycholate, and 0.1% SDS) containing protease and phosphatase inhibitors, and extracted proteins were quantified by Bradford assay. Sixty micrograms of extracted proteins was boiled and electrophoresed for 1.5 h at 150 V on a 12% (wt/vol) polyacrylamide/SDS gel. Samples were transferred for 1.5 h at 100 V to PVDF membranes in transfer buffer (25 mM Tris base, 192 mM glycine, and 20% (vol/vol) methanol). Membranes were blocked in 5% (wt/vol) nonfat dry milk in TBST (10 mM Tris HCl (pH 7.4), 167 mM NaCl, 1% Tween-20) for 1 h and incubated overnight at 4 °C with anti-ERAS rabbit polyclonal antibody or anti-β-actin mouse monoclonal antibody. Blots were washed three times in TBST and subsequently incubated for 1 h at room temperature with horseradish peroxidase (HRP)-conjugated donkey anti-rabbit or donkey anti-mouse secondary antibody diluted 1:3000, in 5% (wt/vol) milk/TBST. The blots were then washed and visualized by enhanced chemiluminescence.

### Immunoprecipitation

Total protein extracts from equine tissues such as the placenta, ovary, testis, kidney, spleen, and liver, obtained as previously described, were immunoprecipitated. Protein samples (500 μg) were incubated with anti-ERAS primary antibodies or with rabbit IgG for 1 h at 4 °C with gentle shaking. Following incubation, centrifugation (1,000 × g, 5 min at 4°C) was carried out, and the samples were collected and incubated overnight with 30 μl of Protein A/G-Plus Agarose (sc-2003) (Santa Cruz Biotechnology, TX, USA) at 4°C. Immunoprecipitates were washed four times in complete lysis RIPA buffer (as described above) and separated on polyacrylamide gels. Following the transfer of proteins, the membranes were blocked for 1 h at room temperature in 5% bovine serum albumin and incubated overnight at 4°C with primary antibodies. After three washes in Tris-buffered saline, the membranes were incubated with the respective secondary antibodies for 1 h at room temperature. Chemiluminescent signals were then developed with Western Blotting Luminol Reagent (Santa Cruz Biotechnology, TX, USA), and detected by the ChemiDoc XRS Plus gel documentation system (Bio-Rad, CA, USA).

### Statistical Analysis

The results are presented as the means ± SE. GraphPad PRISM software version 8 (GraphPad Software, San Diego, CA) was used to assess the expression levels by one-way ANOVA, which was followed by Tukey's test for multiple comparisons of the means. *P*-values ≤ 0.05 were considered statistically significant.

## Results

**Figure 1:**
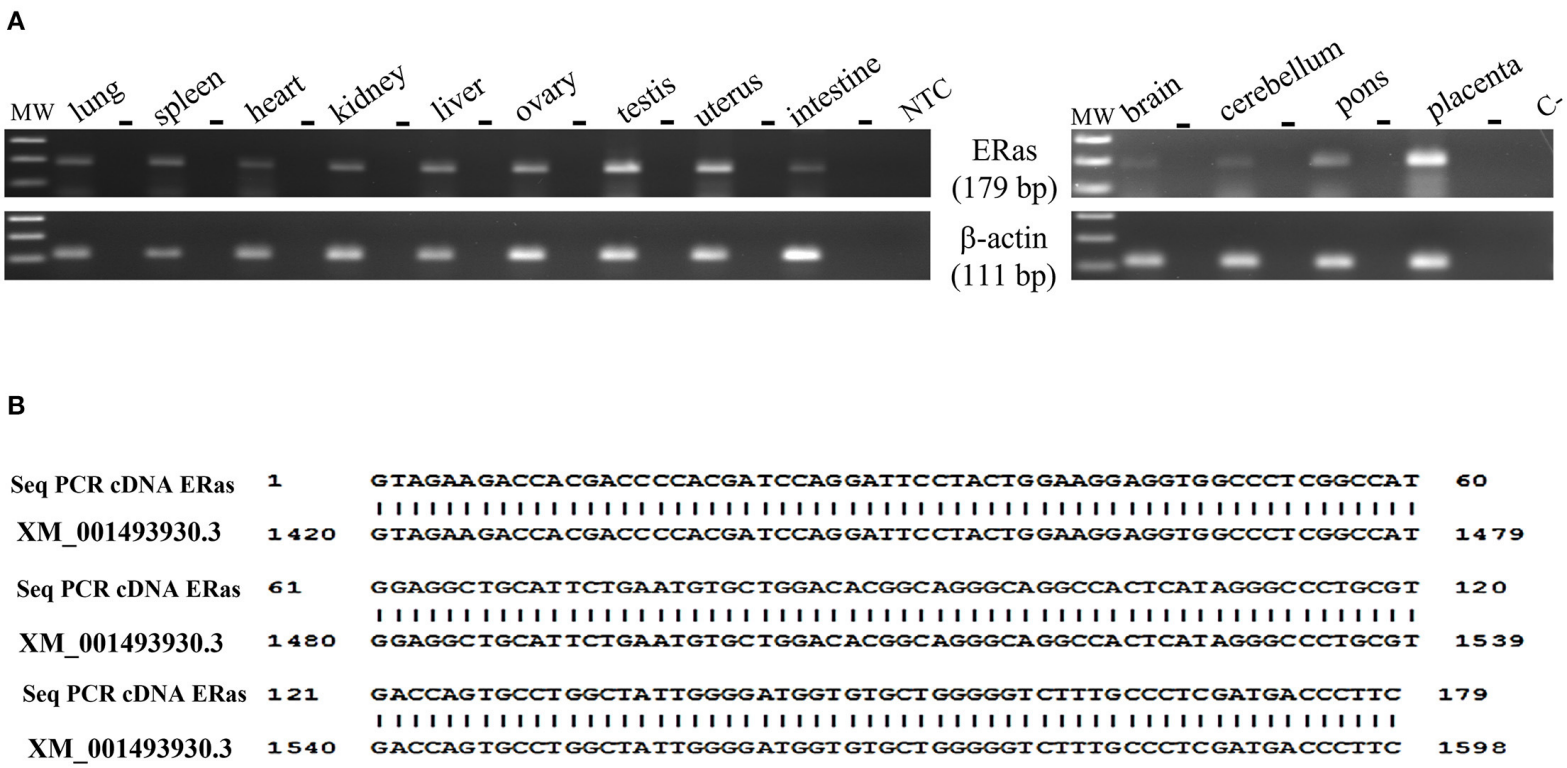
**(A)** Electrophoresis of RT-PCR products for evaluating ERAS mRNA expression in equine tissues. MW: DNA molecular weight marker (100 bp). The PCR was performed both on samples in which reverse transcriptase (RT) was added to the reaction mix and in those without RT (-), using the same amount of RNA. NTC: no template control, omitted RNA template from the reaction mix; C-: PCR negative control. **(B)** 100% identity between the sequence of the amplicons (Seq PCR cDNA ERas) and the sequences of Equus caballus ES cell expressed Ras (ERAS), mRNA Sequence ID: XM_001493930.3.

**Figure 2:**
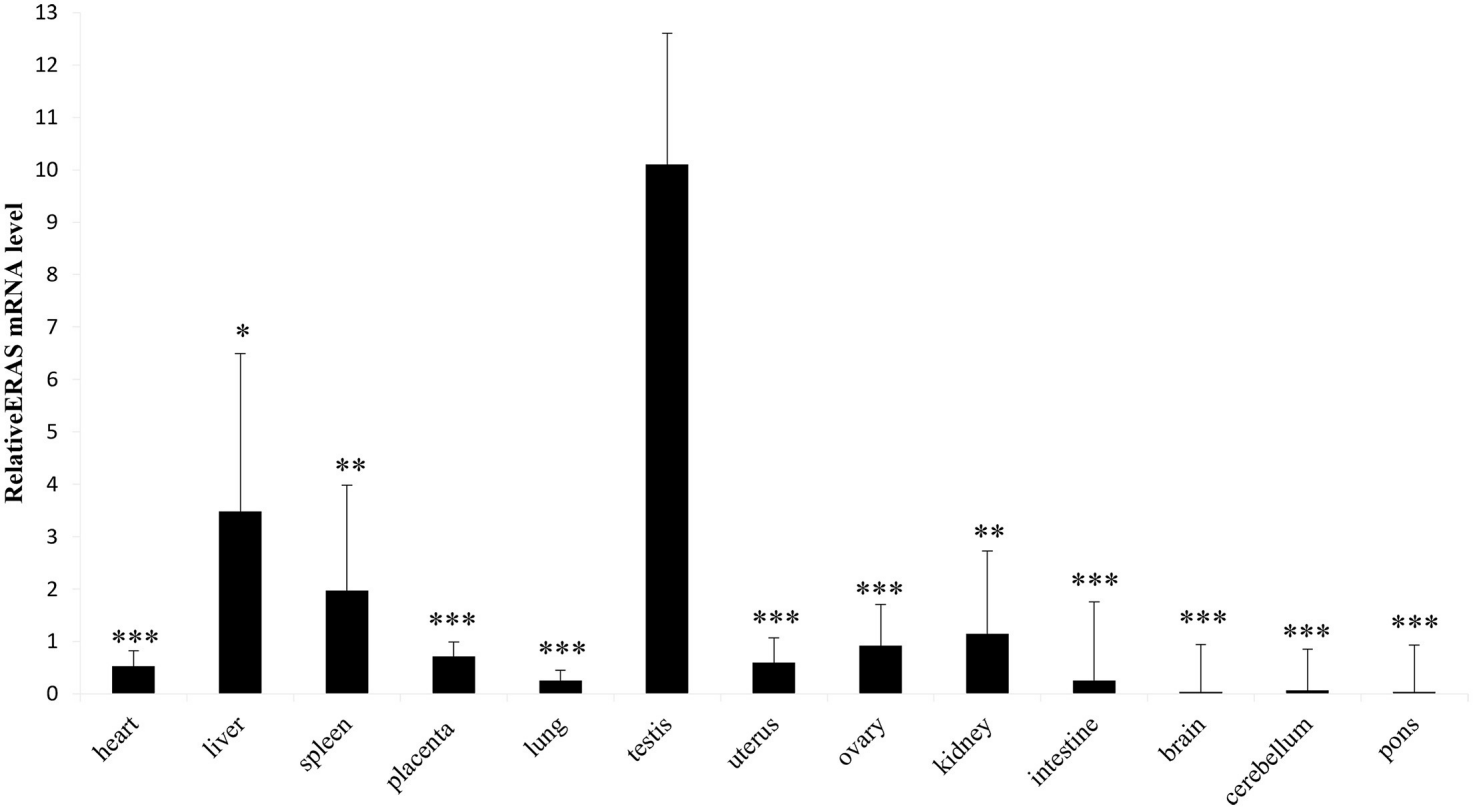
Representative RT-qPCR shows equine tissues ERAS mRNA levels. Data are expressed as mean and standard error mean (S.E.M) of three separate experiments from several animals, each performed in triplicate, related to ERAS levels in the testis (*p < 0.05; **p < 0.01; ***p < 0.001).

**Figure 3:**
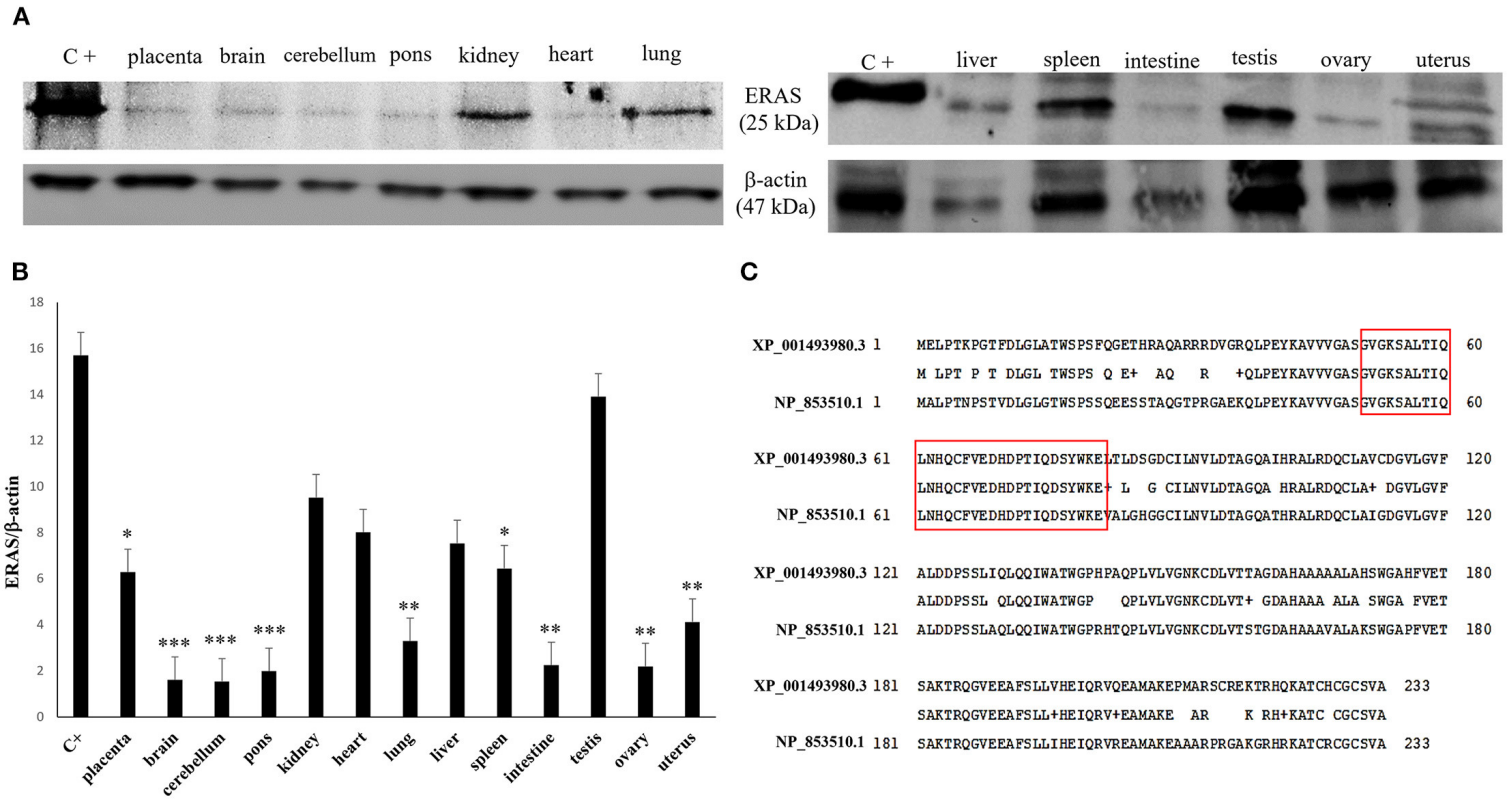
**(A)** Western blot (WB) analysis of ERAS protein in equine tissues. C+: WB positive control, ERAS HEK 293T cell transient overexpression lysate. An expected ~25kDa band was shown in all samples. Actin was used as loading control. **(B)** The values of the densitometric analysis were related to the ERAS protein levels in the positive control (*p < 0.05; **p < 0.01; ***p < 0.001). **(C)** Alignment between the sequences of the human (NP_853510.1) and equine (XP_001493980.3) ERAS protein showed an 81% identity. The amino acid residues of the epitope against the specific antibody used in the current study have underlined in red. This immunogenic portion of ERAS is composed of 31 amino acid residues (51–81 position) showing a 100% identity between the human and horse epitope.

**Figure 4:**
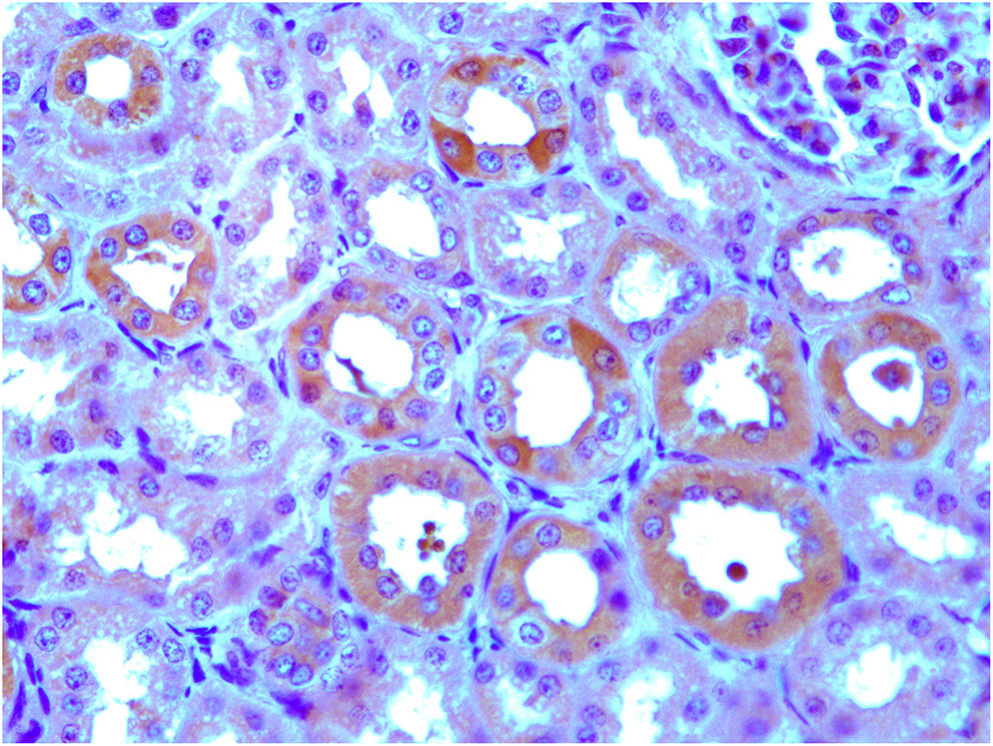
Immunohistochemical evidence of ERAS showing plasmatic and intracytoplasmic localization in epithelial cells of the kidneys and placenta. Inset: No immunoreactivity was seen in renal and placental epithelial cells where the primary anti-ERAS antibody was replaced by appropriate species- and isotype-matched immunoglobulins.

RT-PCR showed amplicons of ERAS cDNA in organs from all examined horses. The presence of ERAS mRNA was confirmed by sequencing the amplicons that showed 100% identity with the RNA sequences of Equus caballus ES cell expressed Ras (ERAS) (mRNA Sequence ID: XM_001493930.3) (Figure 1). The highest ERAS mRNA expression levels were detected in the testicular samples. Significant lower expression levels of ERAS mRNA (^*^ *p* < 0.05 for liver; ^**^p < 0.01 for spleen and kidneys; ^***^p < 0.001 for remaining ones) were detected in all examined organs compared to testicular samples (Figure 2). In particular, the exact *p*-values were reported in Supplemental Table 1. The brain appeared to have limited ERAS mRNA expression. Western blot analysis revealed the presence of protein expression in all examined organs. The testis, liver, kidneys, spleen, placenta showed the highest protein expression levels. Significant lower expression levels were detected in the organs such as the placenta and spleen (*p*-value ^*^p < 0.05), lung, intestine, ovary, uterus (*p*-value ^**^p < 0.01), medulla oblongata, pons and cerebellum (*p*-value ^***^ *p* < 0.001) (Figure 3). The exact *p*-values were reported in Supplemental Table 2. Immunohistochemically, ERAS was found to be localized prevalently in the plasma membrane as well as cytoplasm of the cells (Figure 4).

**Figure 5:**
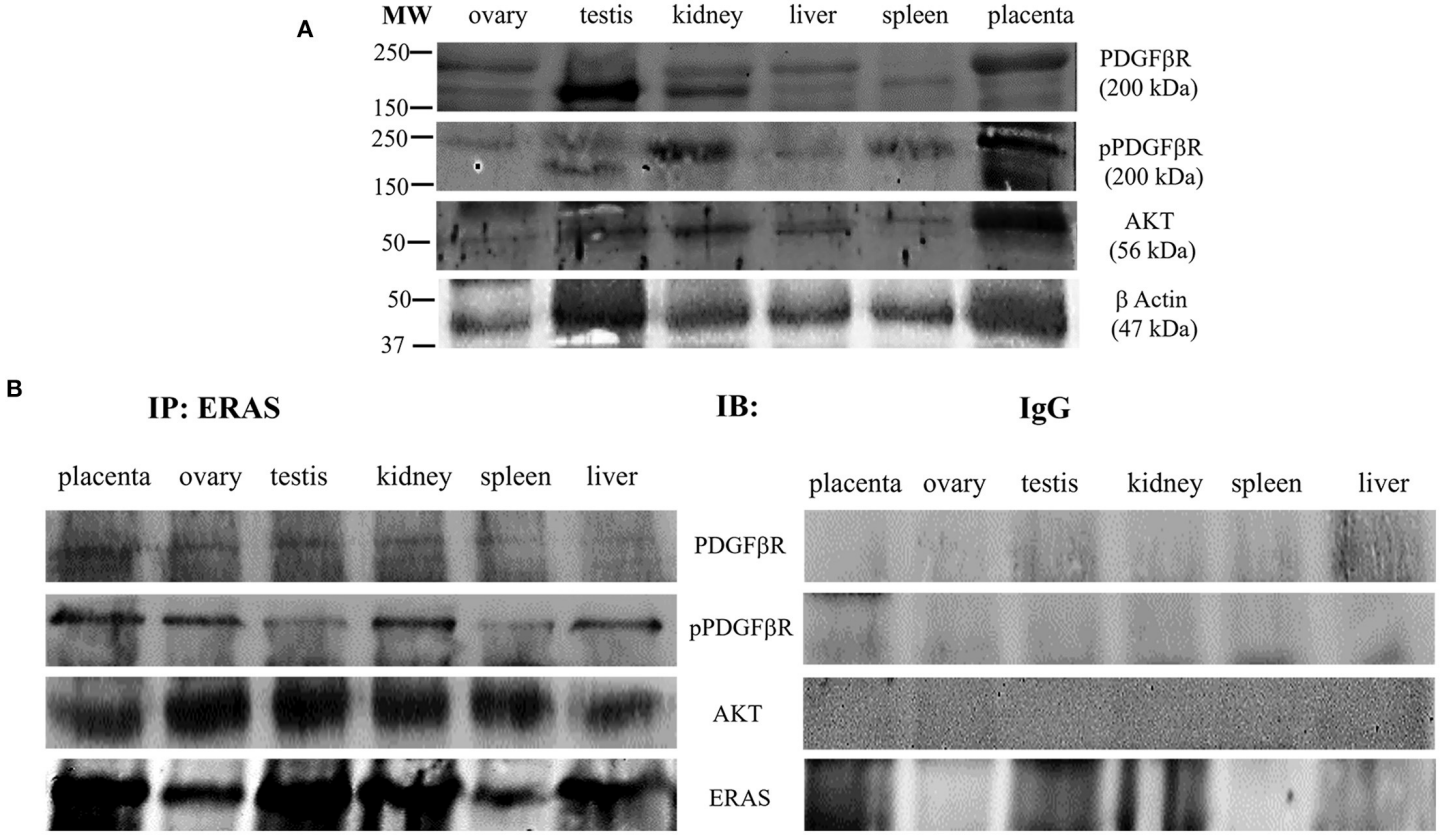
**(A)** Representative WB analysis and **(B)** ERAS immunoprecipitation (IP) of pPDGFβR, total PDGFβR and AKT in equine tissues from several animals. WB analysis performed on IP revealed that ERAS interacted with pPDGFβ, PDGFRβ and AKT.

ERas is a functional gene that contributes to the homeostasis of bovine cells and may be involved in cellular signaling mechanisms under physiopathological conditions (5, 6). Therefore, to investigate the role, if any, of ERAS in normal equine cells, we sought to determine whether ERAS could be associated with factors that modulate cellular signaling. ERAS was found to physically interact with PDGFβR, as shown by immunoprecipitation studies. PDGFβR interacting with ERAS was shown to be highly phosphorylated, which suggested that the receptor was functionally activated. Furthermore, in all tissue samples, we found that ERAS interacted with AKT (Figure 5), an important downstream effector of activated PDGFβR (1, 5).

**Figure 6:**
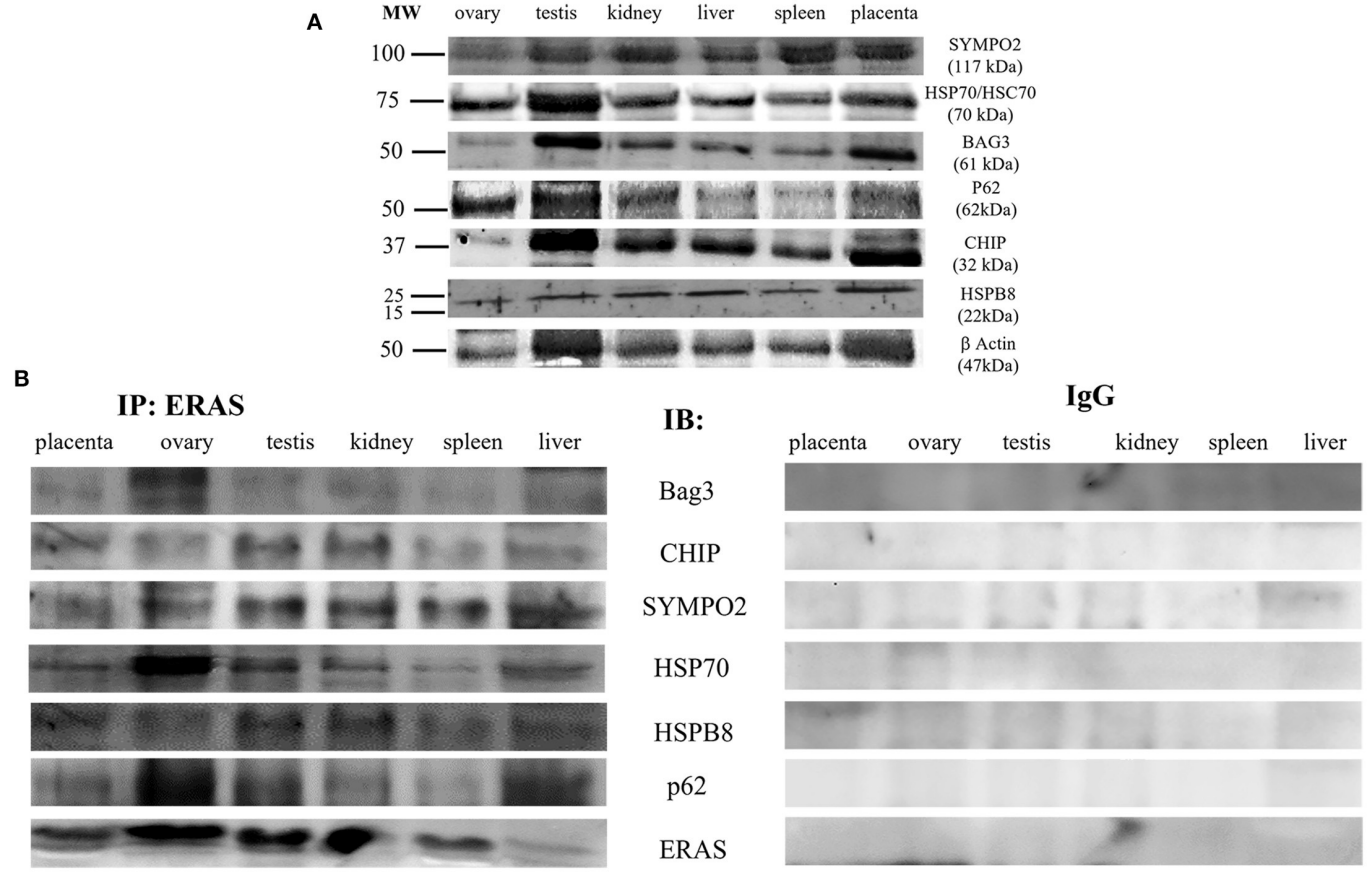
**(A)** Representative WB analysis and **(B)** ERAS immunoprecipitation (IP) in equine tissues from several animals. WB analysis carried out on IP revealed that ERAS interacted with some of proteins involved in autophagy.

**Figure 7:**
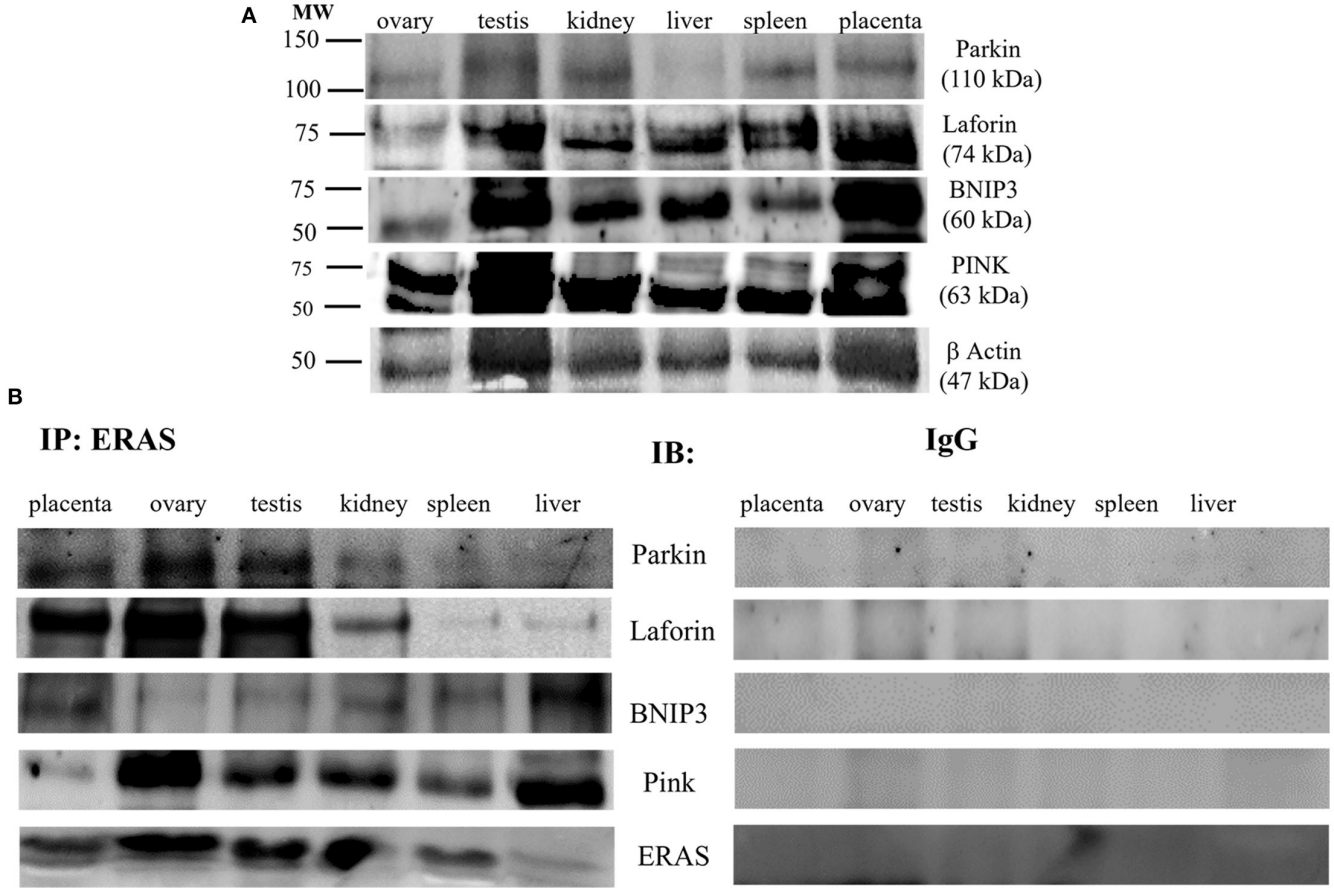
**(A)** Representative WB analysis and **(B)** ERAS immunoprecipitation (IP) in equine tissues from several animals. WB performed on IP revealed that ERAS interacted with some of proteins involved in mitophagy.

It has been suggested that ERAS plays a crucial role in selective macroautophagy (19). Therefore, we investigated whether ERAS might be involved in cellular proteostasis *via* autophagy. ERAS was found to be a physical partner of the cochaperones BAG3, a Bcl-2-associated athanogene protein, and CHIP, a chaperone-associated ubiquitin ligase. ERAS also interacted with the chaperones Hsc70/Hsp70, and HspB8, as well as with Synpo2, a protein essential for autophagosome formation, and the autophagic ubiquitin adaptor p62 (Figure 6). Furthermore, ERAS may be an important factor of both parkin-dependent and parkin-independent activated mitophagy in viral infections (17, 18). Therefore, we sought to investigate the relationship, if any, between ERAS and mitophagy proteins in physiology. We found that ERAS interacted with PINK1, parkin, a ubiquitin E3 ligase, and laforin, which are proteins that play a crucial role in parkin-mediated mitophagy. Furthermore, ERAS was found to be a partner of BNIP3, a multifunctional mitochondrial outer membrane protein known to be an important mitophagy-mediating receptor (Figure 7).

## Discussion

Our study indicates that ERas, a new member of the Ras family, is a functional, constitutively expressed gene in the tissues of adult horses (*Equus caballus*). Usually, ERAS is localized to the plasma membrane (2, 4). The results presented here and those of other studies (6) reveal that ERAS expression can have both membranous and intracytoplasmic patterns in all tissues. It is conceivable that differential posttranslational modifications, including palmitoylation, could be responsible for promoting subcellular compartmentalization similar to other Ras GTPases, which can influence signaling differences (2, 20). It is worth noting that spatial location generates a distinct response, with broader outputs from Golgi-Ras and mitocondrion-Ras (21).

This study demonstrates that ERAS is a partner of numerous interactors that mediate many important signaling pathways crucial for metabolism and cell homeostasis. Therefore, ERAS appears to represent a signaling hub of the protein network known to play a role in fundamental cell processes.

Immunoprecipitation demonstrated that in the tissues of adult horses a ternary complex composed of ERAS, the activated form of PDGFβR and AKT, was present. These findings are consistent with those from experimental studies [(1); 22] and strengthen the molecular findings obtained in tissues from adult cattle under both physiological and pathological conditions (5, 6, 23). It is conceivable that ERAS also plays a crucial role in AKT signaling activation *via* PDGFβR in normal cells of adult horses. It is worth noting that PDGFβR signaling is of physiological importance in adult animals, as AKT signaling is important for many physiological activities of cells in adult tissues. In particular, it has been shown that activation of AKT pathway by PDGFRs promotes actin reorganization, directed cell movements, stimulation of cell growth, and inhibition of apoptosis (24, 25). As pPDGFβR is involved in blood vessel formation, ERAS may play an important role in tissue repair *via* angiogenesis. In addition, ERAS and its downstream AKT signaling pathway are important facilitators of the somatic reprogramming process, which is essential in development and tissue homeostasis (7, 26) and in maintaining quiescence in hepatic stellate cells (HSCs), which have been identified as liver-resident mesenchymal stem cells involved in liver development, immunoregulation, regeneration, and fibrogenesis (22).

The immunoprecipitation findings of the current study showed that ERAS in somatic cells of adult horses is a physical partner of a large network of proteins known to play a role in cellular proteostasis. Indeed, molecular findings showed that ERAS interacted with the molecular chaperones Hsc70/Hsp70 and HSPB8 and the cochaperones BAG3 and CHIP. All these proteins are known to be part of so-called chaperone-assisted selective autophagy (CASA) machinery, also known as BAG3-mediated selective macroautophagy (27), a molecular complex believed to be an adaptive mechanism to maintain cellular homeostasis. Furthermore, p62, and Synpo2 were readily detectable in immunoprecipitated ERAS. The stress-inducible cellular protein p62, functions as a selective autophagy receptor for the degradation of ubiquitinated substrates (28). Synpo2, a cytoskeleton adaptor protein, has been shown to be responsible for autophagosome formation during CASA (28, 29). It has been shown that BAG3 facilitates the functional interplay between Hsc70/Hsp70 and HSPB8 and autophagic degradation of CASA-bound clients via p62. Molecular findings of this study suggest that ERAS could be a player in basal BAG3-mediated selective autophagy, which represents a pivotal adaptive safeguarding and emergency system of protein quality control (PQC), that operates physiologically to ensure cellular proteostasis (30). Our suggestions appeared to be corroborated by previous studies that demonstrated that ERAS plays a crucial role, through BAG3, in selective autophagy induced by BPVs in urothelial cells of cattle (31).

Furthermore, ERAS was shown to interact with a mitophagic network composed of the Parkin/PINK1/BNIP3/Laforin proteins. Damaged mitochondria accumulate PINK1, which then recruits parkin, resulting in ubiquitination of mitochondrial proteins, that are then bound by the autophagic protein p62, thus leading to degradation of mitochondria by mitophagy. In addition, BNIP3, a multifunctional mitochondrial outer membrane protein, harbors an LC3-interacting region (LIR), which strengthens the interaction with light chain3 (LC3) protein, which is involved in mitophagosome formation. Finally, laforin was shown to be a component of a novel pathway of mitophagy mediated by prohibitin 2, an inner mitochondrial membrane (IMM) (18). All these protein interactors found in immunoprecipitated ERAS indicate that ERAS may play an important role in the quality control of mitochondria, which is essential for maintaining cellular homeostasis. It is worth remembering that ERAS has been shown to be involved both in mitophagy mediated by parkin and receptors in naturally occurring papillomavirus-associated bladder tumors in cattle (17, 18).

In conclusion, ERAS is constitutively expressed in tissues of adult horses and its role seems to be critical in the modulation of various biological processes including cell growth, proliferation and immune response. ERAS appears to play a crucial role in the molecular mechanisms mediated by activated PDGFβR and in the BAG3-mediated selective autophagy, which is essential to maintain proteostasis via the dual function of protein transcription and degradation. Therefore, it will be of particular interest to improve our knowledge on the role of ERAS in the homeostasis of cells and tissues of adult horses, particularly those subjected to mechanical tension. The CASA complex appears to be essential for preservation of the cellular structure as well as the cardiovascular, respiratory and urogenital systems (32). Further studies are needed to address ERAS networking in an *in vivo* animal model.

## Data Availability Statement

The original contributions presented in the study are included in the article/Supplementary Material, further inquiries can be directed to the corresponding author.

## Author Contributions

FDe and SR contributed to conception and design of the study. FDe organized the database. AP, NZ, FDel, CD, and IM performed the statistical analysis. SR wrote the first draft of the manuscript. All authors contributed to manuscript revision, read, and approved the submitted version.

## Conflict of Interest

The authors declare that the research was conducted in the absence of any commercial or financial relationships that could be construed as a potential conflict of interest.

## Publisher's Note

All claims expressed in this article are solely those of the authors and do not necessarily represent those of their affiliated organizations, or those of the publisher, the editors and the reviewers. Any product that may be evaluated in this article, or claim that may be made by its manufacturer, is not guaranteed or endorsed by the publisher.
