# Supplementary material for: ERAS Is Constitutively Expressed in the Tissues of Adult Horses and May Be a Key Player in Basal Autophagy
Source: Front Vet Sci. 2022 May 24;9:818294. doi: 10.3389/fvets.2022.818294 (PMC9171053; doi:10.3389/fvets.2022.818294)
Supplement: Supplementary file 1 [file Table_1.DOCX]

Supplemental Table S1 - This table showed the exact p values by real time qPCR of several tissues

| Tissue | P value |
| --- | --- |
| Heart | 0,00095 |
| liver | 0,045 |
| Spleen | 0,087 |
| Placenta | 0,0098 |
| Lung | 0,00084 |
| Uterus | 0,00097 |
| Ovary | 0,00099 |
| Kidney | 0,095 |
| Intestine | 0,00096 |
| Brain | 0,00075 |
| Cerebellum | 0,0068 |
| Pons | 0,00082 |
