# Supplementary material for: ERAS Is Constitutively Expressed in the Tissues of Adult Horses and May Be a Key Player in Basal Autophagy
Source: Front Vet Sci. 2022 May 24;9:818294. doi: 10.3389/fvets.2022.818294 (PMC9171053; doi:10.3389/fvets.2022.818294)
Supplement: Supplementary file 2 [file Table_2.DOCX]

Supplemental Table S2 - This table showed the exact WB p values of several tissues

| Tissue | P value |
| --- | --- |
| Placenta | 0,047 |
| Brain | 0,00087 |
| Cerebellum | 0,00098 |
| Pons | 0,00079 |
| Lung | 0,0096 |
| spleen | 0,035 |
| Intestine | 0,0069 |
| Ovary | 0,0075 |
| Uterus | 0,0067 |
